# Evaluation of the colorimetric malachite green loop-mediated isothermal amplification (MG-LAMP) assay for the detection of malaria species at two different health facilities in a malaria endemic area of western Kenya

**DOI:** 10.1186/s12936-020-03397-0

**Published:** 2020-09-09

**Authors:** James Gachugia, Winnie Chebore, Kephas Otieno, Caroline Wangari Ngugi, Adano Godana, Simon Kariuki

**Affiliations:** 1 Department of Medical Microbiology, College of Health Sciences, Jomo Kenyatta University of Agriculture and Technology, P. O. Box 62000-00200, Nairobi, Kenya; 2 Kenya Medical Research Institute, Centre for Global Health Research, P. O. Box 1578-40100, Kisumu, Kenya; 3 National Malaria Control Programme, Ministry of Health, Kenyatta National Hospital, P. O. Box, Nairobi, 19982-00202 Kenya

**Keywords:** Malaria, *Plasmodium*, Malachite green loop-mediated isothermal amplification, Diagnosis

## Abstract

**Background:**

Prompt diagnosis and effective malaria treatment is a key strategy in malaria control. However, the recommended diagnostic methods, microscopy and rapid diagnostic tests (RDTs), are not supported by robust quality assurance systems in endemic areas. This study compared the performance of routine RDTs and smear microscopy with a simple molecular-based colorimetric loop-mediated isothermal amplification (LAMP) at two different levels of the health care system in a malaria-endemic area of western Kenya.

**Methods:**

Patients presenting with clinical symptoms of malaria at Rota Dispensary (level 2) and Siaya County Referral Hospital (level 4) were enrolled into the study after obtaining written informed consent. Capillary blood was collected to test for malaria by RDT and microscopy at the dispensary and county hospital, and for preparation of blood smears and dried blood spots (DBS) for expert microscopy and real-time polymerase chain reaction (RT-PCR). Results of the routine diagnostic tests were compared with those of malachite green loop-mediated isothermal amplification (MG-LAMP) performed at the two facilities.

**Results:**

A total of 264 participants were enrolled into the study. At the dispensary level, the positivity rate by RDT, expert microscopy, MG-LAMP and RT-PCR was 37%, 30%, 44% and 42%, respectively, and 42%, 43%, 57% and 43% at the county hospital. Using RT-PCR as the reference test, the sensitivity of RDT and MG-LAMP was 78.1% (CI 67.5–86.4) and 82.9% (CI 73.0–90.3) at Rota dispensary. At Siaya hospital the sensitivity of routine microscopy and MG-LAMP was 83.3% (CI 65.3–94.4) and 93.3% (CI 77.9–99.2), respectively. Compared to MG-LAMP, there were 14 false positives and 29 false negatives by RDT at Rota dispensary and 3 false positives and 13 false negatives by routine microscopy at Siaya Hospital.

**Conclusion:**

MG-LAMP is more sensitive than RDTs and microscopy in the detection of malaria parasites at public health facilities and might be a useful quality control tool in resource-limited settings.

## Background

Malaria remains a major public health problem and an impediment to social and economic development, particularly in sub-Saharan Africa. In 2017, of the estimated 219 million cases and 445,000 deaths attributed to malaria worldwide, approximately 90% of cases and deaths were in sub-Saharan Africa [1]. Between 2000–2015, there has been a significant reduction in the global malaria burden, with decline in incidence by 37% and mortality by 60% [1]. However, over the last two years the rate of decline has stalled and even reversed in some regions. This has been attributed to several interconnected challenges including the fact that many people who are infected are not properly diagnosed and therefore do not receive appropriate treatment [2].

Accurate parasitological diagnosis of a malaria case using either quality-assured microscopy or Rapid Diagnostic Tests (RDTs) and prompt treatment with effective artemisinin-based combination therapy (ACT) remains a key strategy in malaria case management and has played a key role in the reduction of the global malaria burden over the last two decades [3]. In addition, pillar 1 of the Global Technical Strategy for Malaria recommends universal access to malaria prevention, diagnosis and treatment for effective disease management and for surveillance [2]. Concerted efforts by national malaria programmes to improve malaria parasitological diagnosis and strategies such as test, treat and track (T3) launched by the World Health Organization (WHO) in 2012 have led to a significant increase in the number of health facilities in sub-Saharan Africa with capacity for microscopy or RDT [4, 5].

However, this is not supported by robust in-country quality assurance/quality control (QA/QC) programmes. This is evident from the findings of several studies that have evaluated the quality of diagnostic capacities in endemic areas, which have reported gaps in malaria microscopy ranging from shortage of trained personnel [6], lack of well-maintained microscopes and quality reagents [7], high workloads [8] and poor performance in species identification and reporting [9]. Similarly, although RDTs are recommended for malaria diagnosis in health facilities where microscopy is not available and at the community level, their performance depends on several factors including; parasite density, patient anti-malarial treatment history, *pfhrp2/3* deletions [10], storage conditions and operator proficiency [11]. Without robust QA/QC systems, these factors could affect the diagnostic performance of RDTs or smear microscopy resulting in erroneous results, poor management of patients, irrational use of anti-malarial drugs and inaccurate surveillance data. Whereas, nucleic acid amplification tests (NAATs) are several orders of magnitude more sensitive than RDTs and microscopy, WHO recommends that NAATs be considered only for epidemiological research and survey mapping of sub-microscopic infections [12]. Nucleic acid amplification tests such as RT-PCR, quantitative nucleic acid sequence-based amplification (QT-NASBA) could be used as reference tests for QA/QC programmes, however, they are prohibitively expensive due to the high cost of equipment required, expensive reagents and the need for highly skilled laboratory personnel [13]. Availability of other NAATs such as the loop-mediated isothermal amplification (LAMP) that do not require thermal cyclers or highly skilled laboratory personnel [14] could be an inexpensive reference test that can be used in a QA/QC programme in resource-limited settings.

In Kenya, parasitological diagnosis of malaria using microscopy or RDTs is recommended for all patients with suspected malaria [15]. Since microscopy is only available at level 3 (health centre) to level 6 facilities (referral hospital), RDTs are used at level 1 (Community Health Workers) and level 2 facilities (dispensaries) or when microscopy is not available at other levels such as when there is power outage or stock out of reagents for microcopy. As in other endemic countries, the Kenya National Malaria Control Programme has developed malaria diagnosis and QA/QC guidelines and manuals [15]. However, implementation has remained a challenge and this is likely to have an impact on patient management and tracking the malaria burden at different levels of the health care system. Additionally, there is limited information on how many patients are missed by routinely performed RDTs and smear microscopy at different levels of the healthcare system.

The main objective of the current study was to compare the performance of routine RDTs and microscopy against an easy-to-use and highly sensitive molecular diagnostic assay, malachite green loop-mediated isothermal amplification (MG-LAMP) at two government health facilities representing different levels of healthcare delivery in Kenya. Although the use of LAMP has been extensively evaluated for malaria diagnosis in areas of low malaria transmission and elimination settings, there is limited information on its use to support a QA/QC system in resource-limited settings.

## Methods

### Study area

The study was conducted at two health facilities (Siaya County Referral Hospital and Rota Dispensary), which are located in a malaria endemic area of western Kenya and serve mostly rural populations. Most of the residents in this area belong to the Luo ethnic group and live in scattered family compounds consisting of one or more houses surrounded by agricultural fields. The main occupation of the residents in this area include subsistence farming, fishing and small-scale trading. The community prevalence of *Plasmodium falciparum* by slide microscopy is 28%, in children aged < 5 years, 42% in the 5 to 15-year old and 18% in those aged > 15 years (KEMRI-CDC unpublished data). Malaria is one of the leading causes of hospital visits and admissions in this area [16]. The primary malaria vectors are *Anopheles gambiae *sensu lato and *Anopheles funestus* and entomologic inoculation rates are < 20 infective bites per person per year [17]. Malaria transmission occurs year-round with two peak seasons from May–July and November–December coinciding with the end of the long and short rains.

**Fig. 1:**
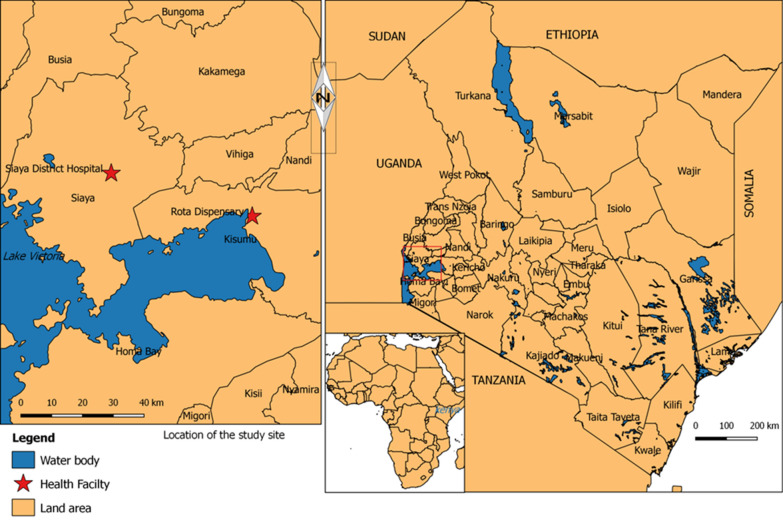
Map showing the location of Rota Dispensary and Siaya County Referral Hospital in western Kenya

Both Rota Dispensary and Siaya County Referral Hospital (Fig. 1) are government-owned and are aligned with the country’s health service delivery system; level-1 (community), level-2 (dispensaries/clinics), level-3 (health centres/maternities/nursing homes), level-4 (sub-county hospitals), level-5 (county referral hospitals) and level-6 (regional and national hospitals). Dispensaries are headed by a nurse and provide promotive and preventive care. Malaria diagnosis at dispensary is primarily by RDT. Country hospitals are headed by a medical officer and undertake mainly curative and rehabilitative services. Malaria diagnosis at this level is performed by laboratory technologists mainly by microscopy, but RDTs can be used if microscopy is unavailable, for example when there is a prolonged power outage or stock outs of reagents for microscopy. Rota Dispensary records 20–30 patients per day mainly for outpatient consultations while complicated cases are referred to levels 3, 4 or 5 facilities. Siaya Hospital outpatient department (OPD) records 40–60 patients per day with both minor and complicated ailments and has inpatient facilities. Any complicated cases are referred to level-6 facilities.

### Study participants

Study participants were recruited at the outpatient departments of the two health facilities if they presented with symptoms suggestive of malaria and were referred to the laboratory for malaria parasitological diagnosis. At Rota Dispensary, RDT (SD Bioline Malaria Ag P.f/Pan 05FK60, Standard Diagnostics, Kyonggi, Republic of Korea) was used for diagnosis. Routine microscopy was used for diagnosis at Siaya Hospital as per the national malaria diagnosis and treatment guidelines. Participants were enrolled into the study after obtaining written informed consent for participants aged over 18 years, parental/caregiver consent for those aged less than 18 years or written assent for emancipated minors. Participants were excluded if they presented with severe disease or reported use of anti-malarial drugs during the past four weeks. Participants found to be malaria positive were treated with artemether-lumefantrine (AL) as per the Kenya Ministry of Health national guidelines.

### Collection of blood samples

Approximately 300 µL of capillary blood was collected into ethylenediaminetetraacetic acid (EDTA) microtainers from enrolled participants. The whole blood was used for preparation of blood smears for expert microscopy, performing malaria RDT, performing MG-LAMP assay and preparation of dried blood spots (DBS) on Whatman® 903 protein saver filter paper (GE Healthcare, USA) for RT-PCR. All samples were assigned a unique study identification number. Blood smears and DBSs were transported to Kenya Medical Research Institute/Centre for Global Health Research (KEMRI/CGHR) malaria laboratories, located about 6.1 km from Rota Dispensary and 56 km from Siaya County Referral Hospital, for storage and analysis.

### Rapid diagnostic tests and malaria microscopy at the health facilities

Rapid diagnostic tests were performed at Rota dispensary using 5 µL of blood according to manufacturer’s instructions. At the Siaya County Referral Hospital, malaria microscopy was performed by a laboratory technologist using the hospital standard operating procedure (SOP) and involved collection of finger prick blood, preparation of thick and thin blood smears using 6 and 2 µL of blood, respectively, staining with 10% Giemsa for 15 min and examination of slides under a microscope. A smear was considered negative if no parasites were detected in 100 high power microscopic fields.

### Expert microscopy at the KEMRI/CGHR malaria laboratories

For endpoint analysis, malaria microscopy was carried out according to WHO basic malaria microscopy guidelines, 2010 [18]. Thick (for parasite density determination) and thin (for *Plasmodium* species identification) smears were prepared from 6 µL and 2 µL of whole blood sample, respectively. The smears were allowed to air dry and then stained with 3% Giemsa for 1 h. The slides were read at 100× objective lens under oil immersion using a compound microscope for determination of both asexual and sexual stage of parasites. A blood smear was considered negative if 100 microscopic high-powered fields showed no parasites. If a blood smear was positive, malaria parasites were counted in 40 microscopic high-powered fields and parasite densities expressed per microlitre (µL). All blood smears were examined independently by two expert microscopists blinded to each other’s results. Where the two readings differed in results (one reader positive and the other negative), parasite species, or if the higher count divided by the lower count was ≥ 2 (for high and medium parasitaemia) and ≥ 10 (for low parasitaemia), smears were re-examined by a tie-breaker microscopist who was blinded to the results of the first two readers. All the microscopists were enrolled and had passed a quarterly external quality assurance programme administered by the National Institute of Communicable Diseases (NICD), South Africa.

### MG-LAMP assay

The MG-LAMP was performed at the health facilities by boil-and-spin method [19] using 50 µL of the collected blood. DNA was released from whole blood by boiling in a heat-block at 95 °C for 10 min. The samples were centrifuged for 3 min at 15,000 × g and the supernatant (containing the DNA) was collected and used for the MG-LAMP assay. Five µL of the supernatant was used in the MG-LAMP assay and the rest stored in the -80ºC freezer. The MG-LAMP assay was performed in a 20 μL total reaction volume containing 2X in-house buffer (40 mM Tris–HCL pH 8.8, 20 mM KCl, 16 mM MgSO_4_, 20 mM (NH_4_)SO_4_, 0.2% Tween-20, 0.8 M Betaine, 2,8 mM of dNTPs each), 0.004% MG, 8 units of *Bst* polymerase (New England Biolabs, Ipswich, MA) and 5 μL of template DNA [20]. Mitochondria *Plasmodium* genus-specific primers were used to amplify the DNA at 63 °C for 60 min using a simple heat block. The samples were allowed to cool for 15 min before being scored by three independent readers by visual inspection of color change. Positive (known *P. falciparum)* and negative (no template/DNA) control samples were included in each run. Positive samples retained a light green/blue malachite green colour while negative samples were colourless. For the purpose of quality control, 20% of the samples were randomly selected and retested at KEMRI/CGHR Malaria Laboratories.

### Quantitative real-time PCR

The QIAamp DNA Mini Blood Kit (Quigen, Valencia, CA) was used to extract DNA from DBS prepared from 50 µL of blood. Commercially available TaqMan Universal Master Mix (Applied Biosystems) was used. Species-specific probes corresponding to *P. falciparum* was used to detect the presence of *P*. *falciparum.* The Rougemont real-time PCR was performed using standard equipment and methods as previously described [21]. Positive (known *P. falciparum* positive sample) and negative (no template/DNA) control sample were included in each run. All samples were run in duplicates. A threshold cycle number (Ct) of 40 was used as the cut-off in order to consider a sample positive or negative: all samples which did not amplify and those that amplified after a Ct value of 40 were considered negative and all samples that amplified before Ct value of 40 were considered positive.

### Statistical analysis

All data was collected using standardized forms and entered into Microsoft Excel (Microsoft Corp, Redmond, Washington, USA). Data analysis was carried out using Stata version 14.2 (StataCorp, College Station, TX, USA). The sensitivity, specificity, positive predictive value (PPV) and negative predictive value (NPV) were determined for rapid diagnostic test (RDT), routine microscopy, expert microscopy and MG-LAMP, using RT-PCR as the reference standard. Sensitivity was calculated as (number of true positives)/(number of true positives + number of false negatives) × 100, and specificity was calculated as (number of true negatives)/(number of true negatives + number of false positives) × 100. The PPV was calculated as (number of true positives)/(number of true positives + number of false positives) × 100, and the NPV was calculated as (number of true negatives)/(number of true negatives + number of false negatives) × 100. Kappa coefficient was calculated to assess the agreement among the different diagnostic methods. P value below 0.05 was considered statistically significant.

## Results

### Characteristics of study participants

**Fig. 2:**
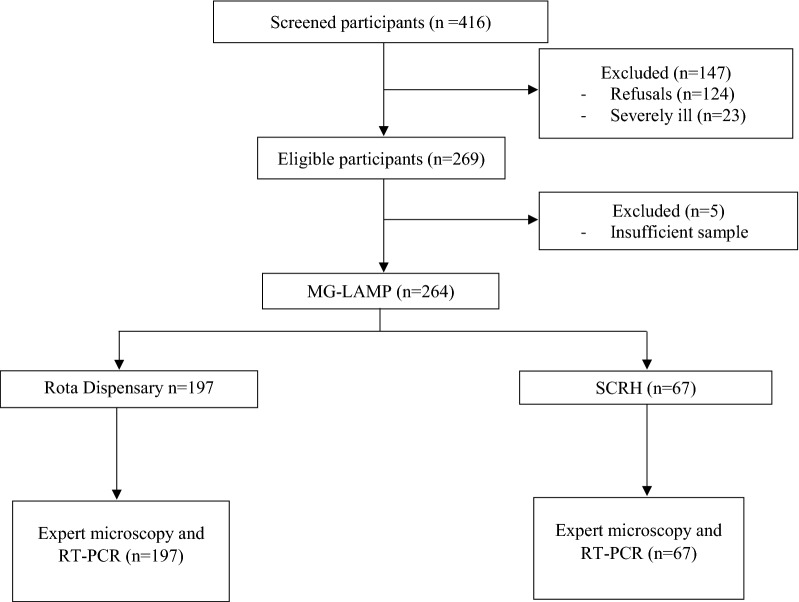
Malaria diagnosis flow chart at the two health facilities. *SCRH* Siaya County Referral Hospital

A total of 416 patients presenting to the two health facilities with suspected malaria were screened for enrollment into the study. Two hundred and sixty-four were enrolled, 197 at Rota Dispensary and 67 at Siaya Hospital and one hundred and fifty-two participants were excluded for various reasons (Fig. 2). The characteristics of the study participants are shown on Table 1. There was no significant difference in gender and mean parasite densities by expert microscopy for participants enrolled at the two health facilities. However, participants enrolled at Rota Dispensary were older, 16.8 years (range 6 months–62 years) compared to those who were enrolled at Siaya Hospital, 7.2 years (range 8 months-–51 years).

**Table 1:** Characteristics of the study participants

Characteristic	Rota dispensary N = 197	Siaya Hospital N = 67	P-value
Gender, n (%)			
Male	71 (36)	28 (42)	0.3809
Female	126 (64)	39 (58)	0.3809
Age, years (R)	16.8 (6 months–62 years)	7.2 (8 months–51 years)	0.0000
Mean parasite density, P/µL (R)	84,638 (0–1,005,163)	69,250 (0–473,175)	0.6422

### Malaria positivity by RDT, routine microscopy, expert microscopy, MG-LAMP and RT-PCR

**Fig. 3:**
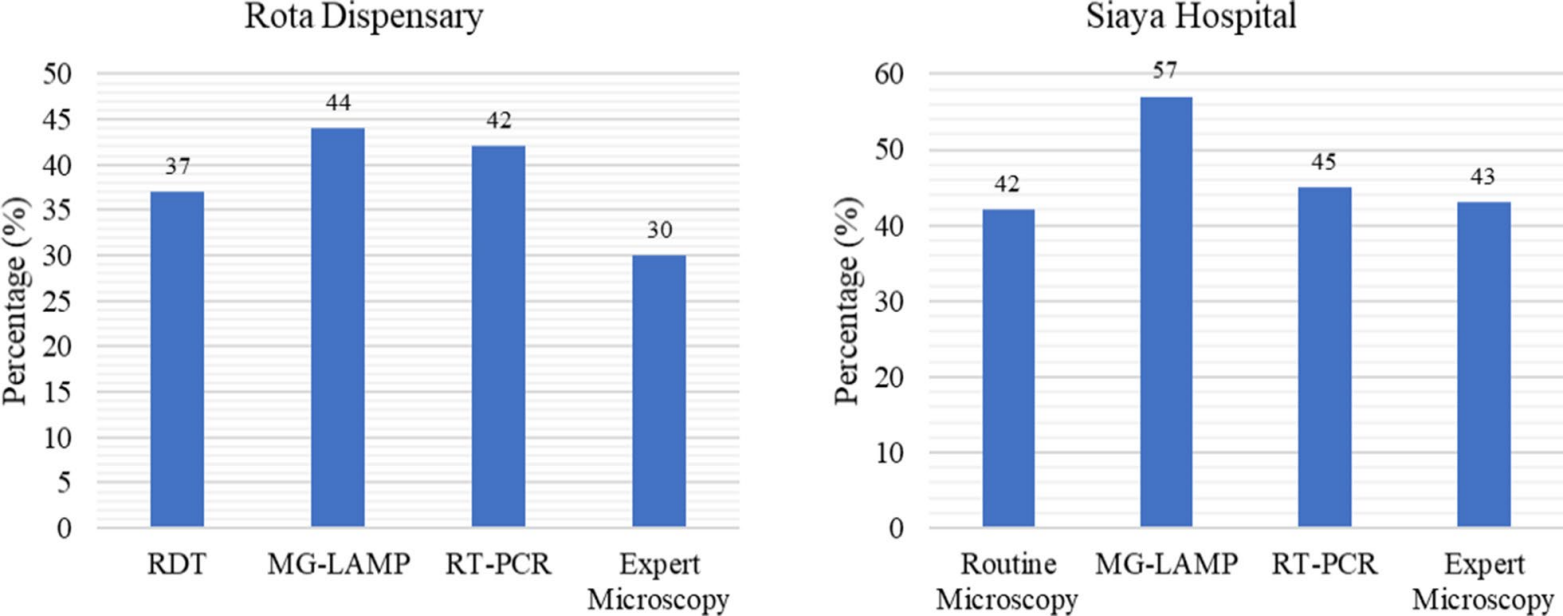
The malaria positivity by RDT, routine microscopy, MG-LAMP, RT-PCR and expert microscopy at Rota Dispensary and Siaya Hospital. Routine microscopy carried out at Siaya Hospital by technicians hired by the hospital, while expert microscopy was carried at KEMRI/CGHR Malaria Laboratories by study staff

At Rota Dispensary, where RDTs are used for malaria diagnosis, the malaria positivity by RDT, MG-LAMP, RT-PCR and expert microscopy were comparable, 37%, 44%, 42% and 30% respectively. Similarly, at Siaya Hospital where microscopy is used for routine diagnosis, there was no significant difference in positivity rate by routine microscopy, MG-LAMP, RT-PCR and expert microscopy (Fig. 3).

### Discordant results between RDT, routine microscopy, expert microscopy, MG-LAMP and RT-PCR

Table 2 shows the number of patients identified as positive or negative by RDT, routine microscopy, expert microscopy, MG-LAMP and RT-PCR at the two health facilities. At Rota dispensary, there were a total of 15 participants who were positive by RDT but negative by MG-LAMP (14), RT-PCR (8) and expert microscopy (15). At the same health facility, a total of 29 participants were positive by MG-LAMP (29), RT-PCR (18) and expert microscopy (2) but negative by RDT. At Siaya Hospital, there was agreement between routine and expert microscopy on the number of positive participants but the results of 3 were discordant and scored as positive by routine microscopy but negative by MG-LAMP (3) and RT-PCR (3). At the same health facility, a total of 13 participants were positive by MG-LAMP (13), RT-PCR (5) and expert microscopy (1) but negative by routine microscopy.

**Table 2:** Summary of discordant results at Rota Dispensary and Siaya County Referral Hospital

Method	RDT positive, n = 72		RDT negative, n = 125	
	Positive (%)	Negative (%)	Positive (%)	Negative (%)
Rota dispensary				
MG-LAMP	58 (80.6)	14 (19.4)	29 (23.2)	96 (76.8)
RT-PCR	64 (88.9)	8 (11.1)	18 (14.4)	107 (85.6)
Expert Microscopy	57 (79.2)	15 (20.8)	2 (1.6)	123 (98.4)

	^a^R-Microscopy Positive, n = 28		R-Microscopy Negative, n = 39	
	Positive (%)	Negative (%)	Positive (%)	Negative (%)
Siaya Hospital				
MG-LAMP	25 (89.3)	3 (10.7)	13 (33.3)	26 (66.7)
RT-PCR	25 (89.3)	3 (10.7)	5 (12.8)	34 (87.2)
Expert Microscopy	28 (100)	0 (0)	1 (2.6)	38 (97.4)

^a^Routine microscopy

### The performance characteristics of RDT, routine microscopy, expert microscopy and MG-LAMP using RT-PCR as a reference

The sensitivity, specificity, positive and negative predictive values of the different diagnostic tests are shown in Table 3. At Rota dispensary, the sensitivity of RDT, MG-LAMP and expert microscopy was 78.1% (CI 67.5–86.4), 82.9% (CI 73.0–90.3) and 72.0% (CI 61–81.3) respectively. At the same facility, the specificity for the three diagnostic tests was 93.0% (CI 86.8–97), 83.5% (CI 75.4–89.8) and 100% (CI 96.8–100) respectively. At Siaya County Referral Hospital, the sensitivity of routine microscopy, MG-LAMP and expert microscopy was 83.3% (CI 65.3- 94.4), 93.3% (CI 77.9–99.2) and 86.7% (CI 69.3–96.2) respectively. At this facility, the specificity for the three diagnostic tests was 91.9% (CI 78.1–98.3), 73.0% (CI 55.9–86.2) and 91.9% (CI 78.1–98.3), respectively.

**Table 3:** Sensitivity, specificity, positive and negative predictive values of RDT, routine Microscopy, MG-LAMP and expert microscopy using RT-PCR as reference

Method	Sensitivity, % (CI)	Specificity, % (CI)	PV, % (CI)	NPV, % (CI)	K-value, % (CI)
Rota dispensary					
RDT	78.1 (67.5–86.4)	93.0 (86.8–97)	88.9 (79.3–95.1)	85.6 (78.2–91.2)	0.72 (0.59–0.86)
MG-LAMP	82.9 (73.0–90.3)	83.5 (75.4–89.8)	78.1 (68.0–86.3)	87.3 (79.6–92.9)	0.66 (0.52–0.80)
E-microscopy	72.0 (61–81.3)	100 (96.8–100)	100 (93.9–100)	83.3 (78–87.6)	0.75 (0.61–0.88)
Siaya Hospital					
R-microscopy	83.3 (65.3–94.4)	91.9 (78.1–98.3)	89.3 (71.8–97.7)	87.2 (72.6–95.7)	0.76 (0.52–1.00)
MG-LAMP	93.3 (77.9–99.2)	73.0 (55.9–86.2)	73.7 (56.9–86.6)	93.1 (77.2–99.2)	0.65 (0.41–0.88)
E-microscopy	86.7 (69.3–96.2)	91.9 (78.1–98.3)	89.7 (74.4–96.3)	89.5 (77.3–95.5)	0.79 (0.55–1.03)

*R-microscopy* routine microscopy, *E-microscopy* expert microscopy, *PPV* positive predictive value, *NPV* negative predictive value, *CI* Confidence Interval

## Discussion

The WHO recommends universal diagnosis of all suspected malaria cases using quality-assured microscopy and RDTs [22]. Due to weak or non-existence of QA/QC systems in endemic areas to support this recommendation, it is important to periodically check the performance of routine diagnostic methods and whether the results compare with those of more sensitive methods such as expert microscopy and molecular methods. In this study, diagnostic results obtained by routine RDTs and smear microscopy at two different levels of health care facilities in an area of high and perennial malaria transmission of western Kenya were compared with results obtained by expert microscopy and more sensitive molecular diagnostic methods; a simple calorimetric-based LAMP and RT-PCR.

There was no significant difference in malaria positivity rate by routine RDT and microscopy at the two health facilities and the comparative diagnostic methods-expert microscopy, RT-PCR and MG-LAMP at the two health facilities. The malaria positivity rate by RDT at Rota Dispensary was slightly higher than the other diagnostics tests used in this study. This is similar to what has been reported in previous studies. In an analysis of 85,000 children enrolled in Demographic and Health Surveys and Malaria Indicator Surveys across 15 countries in sub-Saharan Africa, the mean malaria prevalence was 24.5% by microscopy and 30.3% by RDTs [23]. Similar discrepancies have been shown in São Tomé, 37% by microscopy and 53% by RDT [24]. A study in Tanzania 57.9% malaria positivity by RDT and 52% by microscopy [25], and in Cameroon, 31% by microscopy and 45% by RDT [26]. The higher positivity by RDTs compared to microscopy can be attributed to the fact that HRP-2 based RDTs can remain positive remain positive for longer periods due to persistence of HRP-2 antigens in the blood even after treatment or past infection [27]. This is a challenge for health managers who use results based on RDTs only to estimate malaria case burdens and thus the need for robust secondary diagnostic methods or QA/QC systems in settings where RDTs are the main diagnostic methods. Similar to what has been reported in previous studies that have compared the diagnostic performance of RDTs, routine microscopy and molecular tests, the positivity rates by molecular tests was higher [26–29]. This is not surprising since the threshold of parasite detection for the molecular tests is significantly lower, 1–2 parasites/µL for PCR assays compared to RDTs (100–200 parasites/µL) and microscopy [30].

In this study, 29 and 13 patients at Rota Dispensary and Siaya Hospital, respectively, were negative by the routine diagnostic tests used at these health facilities but they were positive by MG-LAMP, RT-PCR and expert microscopy. This implies that these patients had malaria but were not treated. In the absence of differential diagnosis such as access to blood cultures or PCR to rule out causes of clinical symptoms at many public health facilities in endemic areas, especially in young children, untreated *P. falciparum* malaria can progress rapidly to severe and life-threatening forms of the disease [31]. This can lead to deaths and undermine both the clinical confidence and credibility of health services if patients who might have malaria but are not treated and the cause of symptoms for the hospital visit is not identified [32]. Additionally, untreated cases can contribute to the transmission of malaria in an area [33]. The study also found 15 positive cases by RDT but negative by MG-LAMP, RT-PCR and expert microscopy at the dispensary and 3 cases which were positive by routine microscopy but negative by MG-LAMP and RT-PCR at Siaya Hospital. According to the WHO, both microscopy and RDTs must be supported by a quality assurance programme [34]. This reduces the chance of misdiagnosis and improves patient management. Previous studies have also reported discrepancy between different diagnostic methods [35–37]. Since NAATs diagnostic methods are more sensitive and have a lower limit of parasite detection than RDTs and microscopy, discrepancies are expected in the results obtained by NAATs and other diagnostic methods. Previous studies have reported a limit of detection of < 6 parasites/µL for NAATs compared to 100–200 parasites/µL for RDTs and 50 parasites/µL for microscopy [12, 38]. The sensitivity of MG-LAMP was higher than RDT and microscopy, but a lower specificity at the two health facilities. These results are consistent with previous studies showing the higher sensitivity but lower specificity of MG-LAMP compared to microscopy and RDT [37, 39, 40]. There are many factors which could affect the sensitivity of different diagnostic methods including sample collection method and preparation, efficiency of nucleic acid extraction procedure, amount of blood, amount of template used in the reaction, copy number of target sequence and the buffers, enzymes and other materials used [12]. However, these limitations can be overcome by standardization of the methods and use of quality-assured reagents.

There are several reports highlighting the challenges of malaria diagnostic tests in endemic areas [7–9, 41–43]. Despite these shortcomings, RDTs and microscopy procedures that are not quality assured continue to be used in many malaria endemic areas. This could result in misdiagnosis leading to inappropriate patient management, irrational use of anti-malarial drugs and generation of inaccurate data on the malaria burden. Therefore, there is a need to strengthen the quality assurance processes for malaria diagnostics using inexpensive and novel strategies such as placing simple, inexpensive and more sensitive molecular diagnostic assays at regional centres to strengthen the national QA/QC programmes.

This study has several limitations. There was a selection bias since only patients who presented to the health facilities with symptoms suggestive of malaria were enrolled. However, since the main objective of the study was to evaluate the performance of routine diagnostic methods at two different levels of heath care system, these results could reflect the performance of the diagnostics tests evaluated at health facilities in malaria endemic areas. Another limitation is comparison of results using diagnostic methods that use different input samples, that is, extraction of DNA from DBS versus whole blood. However, these methods are well standardized and are used widely for malaria diagnosis for different objectives such as clinical management-RDTs and microscopy-research and in elimination settings-NAATs. Another limitation is the small sample size at the Siaya Hospital where only 67 participants were enrolled. Patients attending a referral hospital are typically more sick and likely referred from either a dispensary or health centre. Therefore, the results from the dispensary where a larger number of participants were enrolled compensates for the low numbers enrolled at the referral hospital. A major limitation of the LAMP assay is the inability to quantify parasite density. Since the objective of this study was to evaluate whether LAMP can be used as a reference method for RDTs and microscopy in a QA/QC programme, this might not be a major drawback since it is more sensitive than RDTs and microscopy.

## Conclusion

The MG-LAMP evaluated in this study is a simple and sensitive assay for the detection of malaria parasites compared to RDTs and microscopy, which are used for routine malaria diagnosis at health facilities in endemic areas. It could be an ideal and inexpensive reference test in a quality control scheme to monitor the performance of RDTs and smear microscopy in resource-limited settings. This will improve both patient management of suspected malaria cases and the quality of health facility surveillance data.

## Data Availability

The data can be obtained from the corresponding author upon request.

## Abbreviations

DNA: Deoxyribonucleic Acid
EDTA: Ethylene diamine tetra-acetic acid
HRP-2: Histidine Rich Protein-2
JICA: Japan International Cooperation Agency
JKUAT: Jomo Kenyatta University of Agriculture and Technology
KEMRI/CGHR: Kenya Medical Research Institute/Centre for Global Health Research
KU-ERC: Kenyatta University Ethical Review Committee
MG-LAMP: Malachite green-loop-mediated isothermal amplification
NPV: Negative predictive value
P/µL: Parasite per microlitre
PPV: Positive predictive value
QA: Quality assurance
QC: Quality control
RDTs: Rapid diagnostic tests
RT-PCR: Real-time polymerase chain reaction
SERU: Scientific and Ethical Review Unit
WHO: World Health Organization
